# Expert Programmers Have Fine-Tuned Cortical Representations of Source Code

**DOI:** 10.1523/ENEURO.0405-20.2020

**Published:** 2021-01-25

**Authors:** Yoshiharu Ikutani, Takatomi Kubo, Satoshi Nishida, Hideaki Hata, Kenichi Matsumoto, Kazushi Ikeda, Shinji Nishimoto

**Affiliations:** 1 Graduate School of Science and Technology, Division of Information Science, Nara Institute of Science and Technology (NAIST), Ikoma, Nara 630-0192, Japan; 2 Center for Information and Neural Networks (CiNet), National Institute of Information and Communications Technology (NICT), Suita, Osaka 565-0871, Japan

**Keywords:** brain decoding, functional magnetic resonance imaging, program comprehension, programming expertise, the neuroscience of programming

## Abstract

Expertise enables humans to achieve outstanding performance on domain-specific tasks, and programming is no exception. Many studies have shown that expert programmers exhibit remarkable differences from novices in behavioral performance, knowledge structure, and selective attention. However, the underlying differences in the brain of programmers are still unclear. We here address this issue by associating the cortical representation of source code with individual programming expertise using a data-driven decoding approach. This approach enabled us to identify seven brain regions, widely distributed in the frontal, parietal, and temporal cortices, that have a tight relationship with programming expertise. In these brain regions, functional categories of source code could be decoded from brain activity and the decoding accuracies were significantly correlated with individual behavioral performances on a source-code categorization task. Our results suggest that programming expertise is built on fine-tuned cortical representations specialized for the domain of programming.

## Significance Statement

The expertise needed for programming has attracted increasing interest among researchers and educators in our computerized world. Many studies have demonstrated that expert programmers exhibit superior behavioral performance, knowledge structure, and selective attention, but how their brain accommodates such superiority is not well understood. In this article, we have recorded brain activities from subjects covering a wide range of programming expertise. The results show that functional categories of source code can be decoded from their brain activity and the decoding accuracies on the seven brain regions in frontal, parietal, temporal cortices are significantly correlated with individual behavioral performances. This study provides evidence that outstanding performances of expert programmers are associated with domain-specific cortical representations in these widely distributed brain areas.

## Introduction

Programming expertise is one of the most notable capabilities in the current computerized world. Since human software developers keep playing a central role in software projects and directly impact their success, this relatively new type of expertise is attracting increasing attention from modern industries (Li et al., 2015; Baltes and Diehl, 2018) and educational institutes (Heintz et al., 2016; Papavlasopoulou et al., 2018). Moreover, huge productivity variations were repeatedly found even between programmers with the same level of experience (Boehm and Papaccio, 1988; DeMarco and Lister, 2013). Previous studies have shown the psychological characteristics of expert programmers in their behaviors (Vessey, 1985; Koenemann and Robertson, 1991), knowledge structures (Fix et al., 1993; Von Mayrhauser and Vans, 1995), and eye movements (Uwano et al., 2006; Busjahn et al., 2015). Although these studies clearly illustrate the behavioral specificity of expert programmers, it remains unclear what neural bases differentiate expert programmers from novices.

Recent studies have investigated the brain activity of programmers using functional magnetic resonance imaging (fMRI). Siegmund et al. (2014, 2017) contrasted brain activity during program output estimations against syntax error searches and showed that the processes of program output estimations activated left-lateralized brain regions, including the middle frontal gyrus (MFG), inferior frontal gyrus (IFG), inferior parietal lobule (IPL), and middle temporal gyrus (MTG). Their results suggested that program comprehension is associated with natural language processing, division of attention, and verbal/numerical working memory. Peitek et al. (2018a) reanalyzed the same data as Siegmund et al. (2014) to investigate the correlation between the BOLD activation strength and individual programming experience, which was determined by subject’s self-estimation, but did not find any significant trend. An exploratory study argued that a correlation exists between activity pattern discriminability and subjects’ grade point average (GPA) scores counting only courses from the Computer Science department as a proxy for programming expertise (Floyd et al., 2017). However, the GPA scores would reflect a mixture of diverse factors (IQ, memory ability, calculation skills, etc.), and the assumed relationship to programming expertise was difficult to be empirically validated. Further, the main limitation of these prior studies is the use of a homogeneous subject group that only covered a small range of programming expertise. Recruitment of more diverse subjects in terms of their programming expertise may enable the elucidation of the potential differences of brain functions related to the expertise.

Here, we aim to identify the neural bases of programming expertise that contribute to the outstanding performances of expert programmers. To do this, we defined two fundamental factors in our experiment: an objectively determined reference of programming expertise and a laboratory task that exhibits experts’ superior performances under the general constraints of fMRI experiments. First, we adopted the programmers’ ratings in competitive programming contests (AtCoder; https://atcoder.jp/), which are objectively determined by the relative positions of their actual performances among thousands of programmers. We recruited top-rated and middle-rated programmers as well as novice controls to cover a wide range of programming expertise in our fMRI experiment. Second, we developed the program categorization task and confirmed that behavioral performances of this task were significantly correlated with the adopted reference of programming expertise. This confirmation allows us to expect an association between the outstanding performances of expert programmers and brain activity patterns recorded by fMRI while they performed this laboratory task.

Our core hypothesis is that higher programming expertise and experts’ outstanding performances relate to specific multivoxel pattern representations, potentially influenced by their domain-specific knowledge and training experiences. This hypothesis is motivated by prior studies that contrasted multivoxel activity patterns of experts against novices and demonstrated that domain-specific expertise generally associates with representational changes in the brain (de Borst et al., 2016; Martens et al., 2018; Gomez et al., 2019). For example, Bilalić et al. (2016) showed that the multivoxel patterns in expert radiologists’ fusiform face area were more sensitive in differentiating x-ray images from control stimuli than novices. Similarly, identifying the multivoxel pattern representations specific to expert programmers offers a good starting point for understanding the cognitive mechanisms behind programming expertise. From the previous studies on non-expert programmers and expertise in other domains, the high-level visual and left fronto-parietal regions might be inferred as potential neural correlates of programming expertise (Siegmund et al., 2014; Bilalić, 2017). However, to the best of our knowledge, there is no prior evidence that directly associates programming expertise with specific brain regions. Thus, we employ a whole-brain searchlight analysis (Kriegeskorte et al., 2006) to identify the regions related to programming expertise.

## Materials and Methods

### Subjects

To begin this study, we defined three recruiting criteria: *expert*, top 20% rankers in AtCoder who had an AtCoder rate equal to or higher than 1200; *middle*, 21–50% rankers who had an AtCoder rate between 500 and 1199; *novice*, subjects who had four years or less programming experience and no experience in competitive programming. We shared our recruiting message via mailing lists and messaging applications with diverse graduate or undergraduate student communities in Japan. Through this procedure, 95 programmers from 28 universities and three companies completed our entry questionnaire to be registered as candidate subjects. The list of candidate subjects consisted of 19 experts (all male), 43 middles (one female), and 33 novices (nine females). Nine left-handed subjects and 20 subjects with less than half a year experience in Java programming were excluded from the list. Five subjects aged under 20 years old were also excluded to avoid additional bureaucratic processes. We asked the remaining candidate subjects for experiment participation basically on first-in-first-out strategy. Note that setting *novice* as programmers who had an AtCoder rate under 500 was another potential recruiting criterion, but we did not adopt the criterion because low values in the rate reflects two indistinguishable factors: low programming expertise or not enough contest participation. In addition, possession of AtCoder rate itself could imply possession of moderate programming expertise. Thus, our recruiting criteria set *novice* as a programmer with shorter experience in programming and no experience in competitive programming.

Thirty healthy subjects (two females, aged between 20 and 24 years) with normal or corrected-to-normal vision participated in the experiment (for the demographic information of recruited subjects, see Table 1). All were right-handed [assessed by the Edinburgh Handedness Inventory (Oldfield, 1971); laterality quotient = 83.6 ± 24.0, ranged between +5.9 and +100] and understood basic Java grammars with at least half a year experience in Java programming. The averaged AtCoder rates (1967 in *expert* and 894 in *middle*) were equivalent to the top 6.5% and 34.1% positions among 7671 registered players based on the ranking on July 1, 2017, respectively. Seven additional subjects were scanned but not included in the analysis because one (novice) showed neurologic abnormality in MRI images, three (one expert and two middles) retired from the experiment without full completion, three (one expert and two novices) showed strongly-biased behavioral responses judged when the behavioral performance of one or more choices did not reach chance-level in the training experiments, signaling a strong response bias of sticking to a specific choice. This study was approved by the Ethics Committees of Nara Institute of Science and Technology and CiNet and subjects gave written informed consent for participation. The sample size was chosen to match previous fMRI studies on human expertise with similar behavioral protocols (Amalric and Dehaene, 2016; Bilalić et al., 2016; de Borst et al., 2016).

**Table 1:** Demographic information of recruited subjects

	*N*	Sex (M/F)	Age	AtCoder rate	PE (year)	JE (year)	CPE (year)
Expert	10	10 / 0	22.6 ± 1.1	1969 ± 467	6.9 ± 2.8	2.8 ± 2.4	4.1 ± 2.6
Middle	10	9 / 1	22.5 ± 0.8	894 ± 175	4.8 ± 1.7	1.1 ± 0.8	1.3 ± 0.8
Novice	10	9 / 1	21.7 ± 1.2	NA	2.8 ± 0.6	1.4 ± 1.0	NA

Numerics from fourth (age) to last columns denote mean ± SD. PE, programming experience; JE, JAVA experience; CPE, competitive programming experience. Significant differences were observed between PE of expert-novice, middle-novice; CPE of expert-middle (two-sample *t* test, *p* < 0.05 FDR-corrected).

### Stimuli

**Figure 1.**
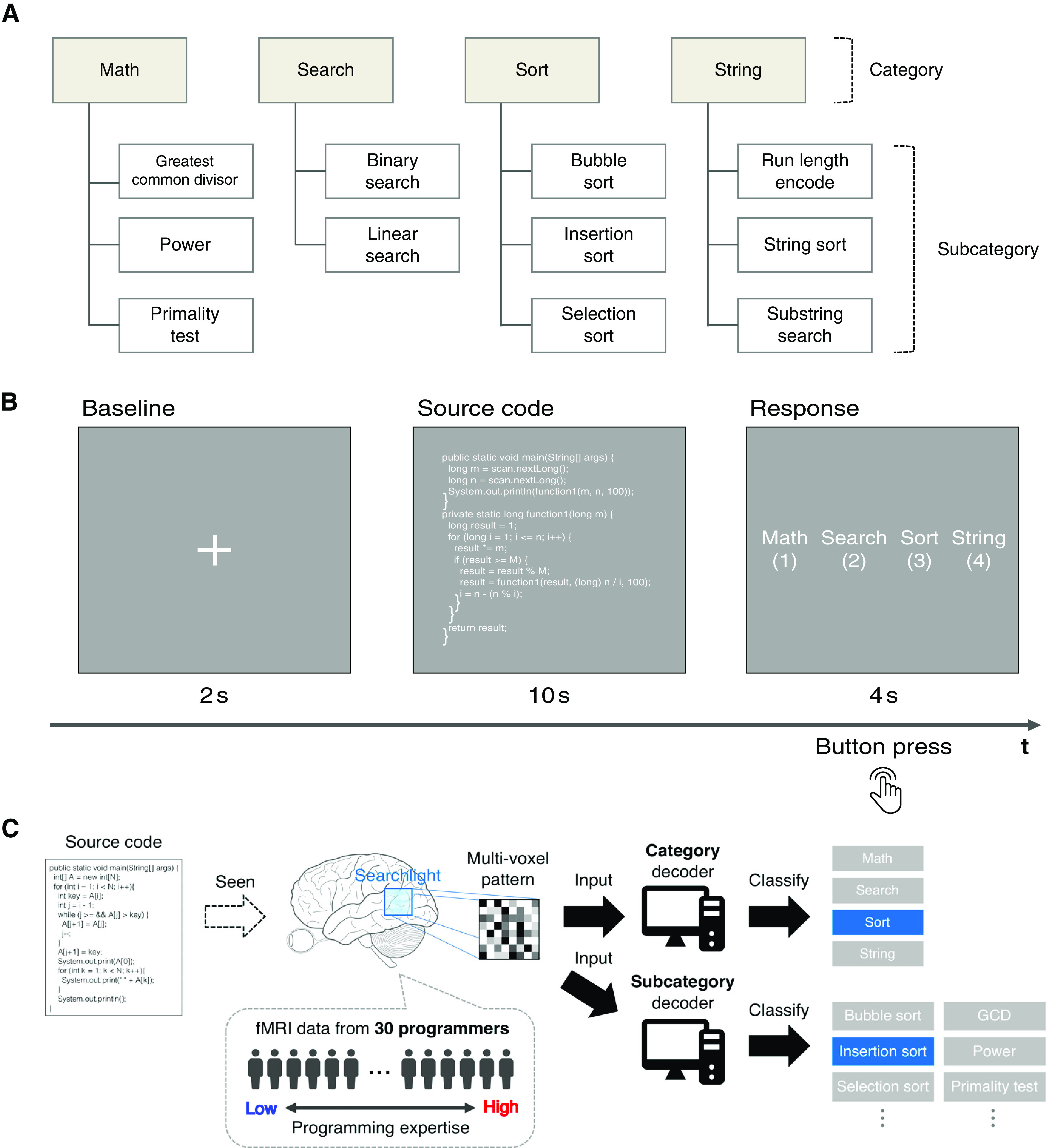
Experimental design. ***A***, Hierarchy of categories used in this study. Category and subcategory represent abstract functionality and concrete algorithms, respectively, based on two popular textbooks of programming. Every code snippet used in this study belonged to one subcategory class and its corresponding category class. ***B***, Program categorization task. After a fixation-cross presentation for 2 s, a Java code snippet was displayed for 10 s in white text without any syntax highlight. Then, subjects responded the category of given code snippet by pressing a button. ***C***, Overview of the decoding framework. MRI data were collected from 30 subjects with different levels of programming expertise while they performed the program categorization task. Whole-brain searchlight analysis (Kriegeskorte et al., 2006) was employed to explore the potential loci of programming expertise. For each searchlight location, a linear-kernel SVM classifier (decoder) was trained on multivoxel patterns to classify category or subcategory of given Java code snippets.

For this study 72 code snippets written in Java were collected from an open codeset provided by AIZU ONLINE JUDGE (http://judge.u-aizu.ac.jp/onlinejudge/); an online judge system where many programming problems are listed and everyone can submit their own source code to answer those problems online. We selected four functional categories (*category*) and eleven subordinate concrete algorithms (*subcategory*) based on two popular textbooks about computer algorithms (Cormen et al., 2009; Sedgewick and Wayne, 2011; for the detailed descriptions, see Fig. 1*A*, Extended Data Fig. 1-1). We first searched in the open codeset for Java code snippets implementing one of the selected algorithms and found 1251 candidates. The reasons why we focused on Java in this study were because the language has been one of the most famous programming languages and prior fMRI studies on programmers also used Java code snippets as experimental stimuli (Siegmund et al., 2014, 2017; Peitek et al., 2018a). To meet the screen size constraint in the MRI scanner, we excluded code snippets with a number of lines of >30 and a max number of characters per line of >120. From all remaining snippets, we created a set of 72 code snippets with minimum deviations of these numbers of lines and characters to minimize visual variation as experimental stimuli; the mean and SD of the number of lines and max characters per line were 26.4 ± 2.4 and 59.3 ± 17.1, respectively. In the codeset, 18 snippets each belonged to one of the category classes and six snippets each belonged to one of the subcategory classes except for the linear search class with twelve snippets (for detailed statistics on each category and subcategory class, see Extended Data Fig. 1-2). The indentation styles of code snippets were normalized by replacing a tab-space with two white-spaces and user-defined functions were renamed to neutral such as “function1” because some of the functions indicated their algorithms explicitly (for example snippets, see Extended Data Fig. 1-3). We verified all code snippets had no syntax error and run correctly without run-time error.

10.1523/ENEURO.0405-20.2020.f1-1 Extended Data Figure 1-1 Description of categories and subcategories provided to subjects in the experiment. Download Figure 1-1, XLSX file.

**Figure 2.**
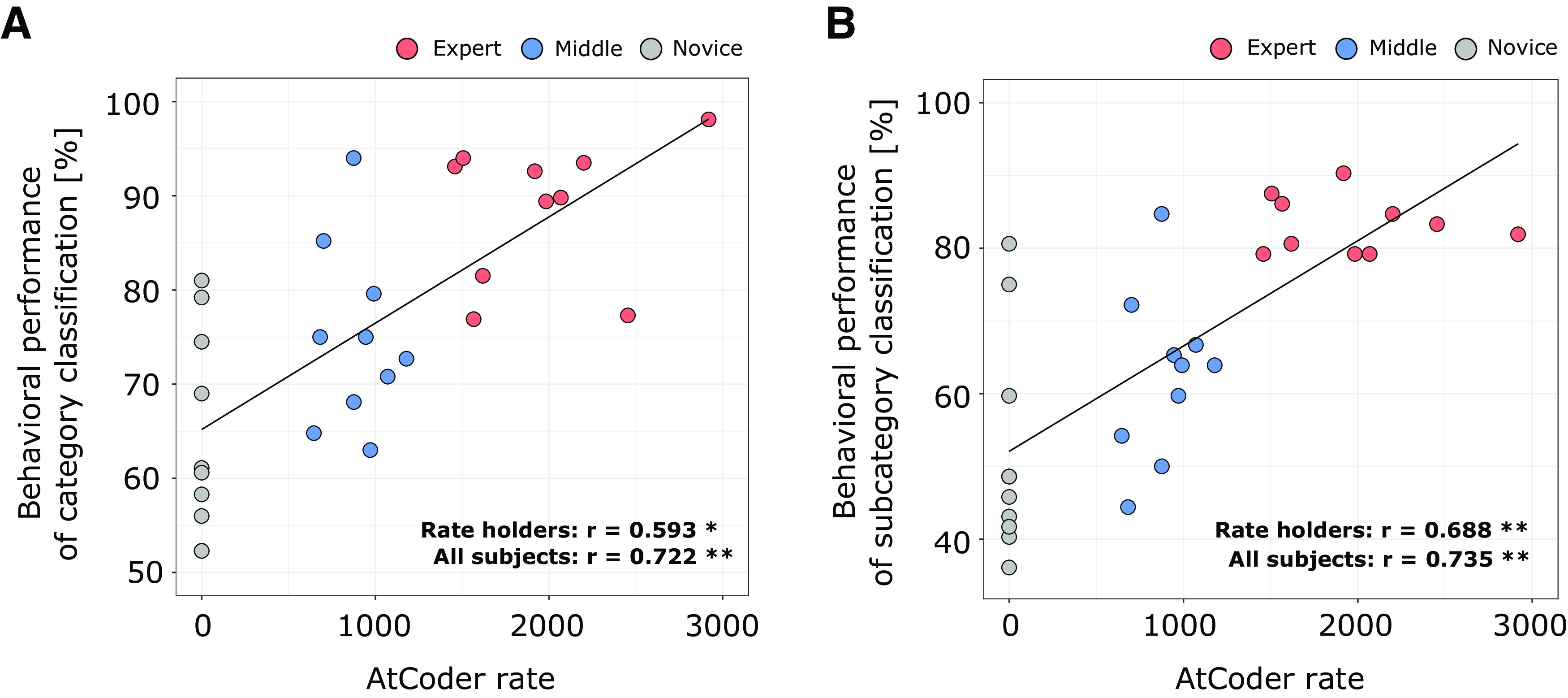
Correlations between behavioral performance and programming expertise indicator. ***A***, Scatter plot of behavioral performances of category classifications against the values of adopted expertise reference (i.e., AtCoder rate). ***B***, Scatter plot of behavioral performances of subcategory classifications against the values of the same expertise reference. Each dot represents an individual subject. Significance of the correlation coefficients (*r*) was denoted as *p < 0.05 and **p < 0.005. The solid lines indicate a fitted regression line estimated from all subject data.

10.1523/ENEURO.0405-20.2020.f1-2 Extended Data Figure 1-2 Statistics of Java code snippets for each category and subcategory class. Numerics from 3rd (LOC) to last columns denote mean ± SD. LOC, lines of code; CPL, max number of characters per line. Download Figure 1-2, XLSX file.

**Figure 3.**
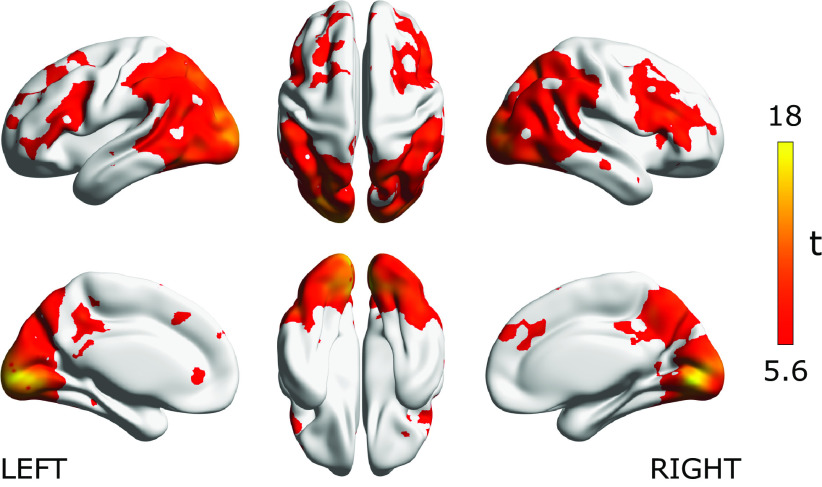
Decoding accuracy for functional category of source code. Significant searchlight locations estimated from all subject data (*N* = 30). Heat colored voxels denote the centers of searchlights with significant decoding accuracy (voxel-level *p* < 0.05, FWE corrected). See Extended Data Figure 3-1 for the distribution of voxel-level peak decoding accuracies. The brain surface visualizations were performed using BrainNet viewer, version 1.61 (Xia et al., 2013).

10.1523/ENEURO.0405-20.2020.f1-3 Extended Data Figure 1-3 Java code snippets used in the study. A total of 72 types of Java code snippet were used in this study. Each belonged to one subcategory and its corresponding category shown in Figure 1*A*. This figure shows example snippets for each subcategory class. Download Figure 1-3, EPS file.

### Experimental design

The fMRI experiment consisted of six separate runs (9 min 52 s for each run). Each run contained 36 trials of the program categorization task (Fig. 1*B*) plus one dummy trial to avoid the undesirable effects of MRI signal instability. We used 72 code snippets as stimuli and each snippet was presented three times through the whole experiment (216 trials in total), but the same snippet appeared only once in a run. We employed PsychoPy (version 1.85.1; Peirce, 2007) to display the code snippets in white text and a gray background without syntax highlighting to minimize visual variations. In each trial of the program categorization tasks, a Java code snippet was displayed for 10 s after a fixation-cross presentation for 2 s. Subjects then responded within 4 s by pressing buttons placed under the right hand to indicate which category class was most plausible for the code snippet and all response data were automatically collected for the calculation of individual behavioral performance. To clarify classification criteria, a brief explanation about each category class was provided before the experiment started. The presentation order of the code snippets was pseudo-randomized under balancing the number of exemplars for each category class across runs. The corresponding buttons for each answer choice were also randomized across trials to avoid linking a specific answer choice with a specific finger movement. Subjects were allowed to take a break between runs and to quit the fMRI experiment at any time.

All subjects took two additional sessions, named “training” and “post-MRI,” outside of the MRI scanner using a laptop computer and PsychoPy to display source code stimuli. The training session was performed within 10 d before the fMRI experiment to mitigate potential confounds caused by task unfamiliarity. The session consisted of three separate runs with the same program categorization task as the fMRI experiment. A different set of 72 Java code snippets from those used in the MRI experiment, which covered the same algorithms, was used as stimuli in the training session; each snippet was presented once or twice in the entire session but the same snippet did not appear twice in a run. The post-MRI session was performed within 10 d after the fMRI experiment for assessment of individual ability in subcategory categorizations and was consisted of two separate runs using the same codeset as the fMRI experiment. Before the post-MRI session started, we explained the existence of subcategory to the subjects and assessed whether they recognized subcategory classes during the fMRI experiment using a questionnaire. Program categorization tasks in the post-MRI session followed the same procedure as the fMRI session. In each trial, a Java code snippet was displayed for 10 s after a fixation-cross presentation for 2 s. Then, within 4 s, the subjects were asked to classify the given code snippet from two or three choices of subcategory classes according to its superordinate category, e.g., “bubble sort,” “insertion sort,” and “selection sort” were displayed when the snippet in “sort” category was presented. We calculated behavioral performance as the ratio of correct-answer-trials to all-trials; unanswered trials, i.e., no button input within the response phase, were regarded as “incorrect” for this calculation. Chance-level behavioral performance was 25% in the training sessions and fMRI experiments and 37.25% in the post-MRI sessions adjusted for imbalanced numbers of answer choices. Again, these two additional sessions were performed outside of the MRI scanner, in other words, every subject did only one experiment with fMRI scanning.

### MRI data acquisition

MRI data were collected using a 3-Tesla Siemens MAGNETOM Prisma scanner with a 64-channel head coil located at CiNet. T2*-weighted multiband gradient echo-EPI sequences were performed to acquire functional images covering the entire brain [repetition time (TR) = 2000 ms, echo time (TE) = 30 ms, flip angle = 75°, field of view (FOV) = 192 × 192 mm^2^, slice thickness = 2 mm, slice gap = 0 mm, voxel size = 2 × 2 × 2.01 mm^3^, multiband factor = 3]. A T1-weighted magnetization-prepared rapid acquisition with a gradient-echo sequence was also performed to acquire fine-structural images of the entire head (TR = 2530 ms, TE = 3.26 ms, flip angle = 9°, FOV = 256 × 256 mm^2^, slice thickness = 1 mm, slice gap = 0 mm, voxel size = 1 × 1 × 1 mm^3^).

### MRI data preprocessing

We used the Statistical Parametric Mapping toolbox (SPM12; http://www.fil.ion.ucl.ac.uk/spm/) for preprocessing. The first eight scans in dummy trials for each run were discarded to avoid MRI signal instability. The functional scans were aligned to the first volume in the fourth run to remove movement artifacts. They were then slice-time corrected and co-registered to the whole-head T1 structural image. Both anatomic and functional images were spatially normalized into the standard Montreal Neurologic Institute 152-brain average template space and resampled to a voxel size of 2 × 2 × 2 mm^3^. MRI signals at each voxel were high-pass2013filtered with a cutoff period of 128 s to remove low-frequency drifts. A thick gray matter mask was obtained from the normalized anatomic images of all subjects to select the voxels within neuronal tissue using the SPM Masking Toolbox (Ridgway et al., 2009). For each subject independently, we then fitted a general linear model (GLM) to estimate voxel-level parameters (*β*) linking recorded MRI signals and conditions of source code presentations in each trial. The fixation and response phases in each trial were not explicitly modeled. The model also included motion realignment parameters to regress-out signal variations because of head motion. Finally, 216 β estimate maps (36 trials × six runs) per subject were yielded and used as input for the following multivariate pattern analysis.

### Multivoxel pattern analysis

We used whole-brain searchlight analysis (Kriegeskorte et al., 2006) to examine where significant decoding accuracies exist using the Decoding Toolbox (version 3.99; Hebart et al., 2015) and LIBSVM (version 3.17; Chang and Lin, 2011). A four-voxel-radius sphered searchlight, covering 251 voxels at once, was systematically shifted throughout the brain and decoding accuracy was quantified on each searchlight location. A linear-kernel support vector machine (SVM) classifier was trained and evaluated using a leave-one-run-out cross-validation procedure, which iteratively treated data in a single run for testing and the others for training. In each fold, training data were first scaled to zero-mean and unit variance by z-transform and test data were scaled using the estimated scaling parameters. We then applied outlier reduction using [−3, +3] as cutoff values and all scaled signals larger than the upper cutoff or smaller than the lower cutoff were set to the closest value of these limits. The SVM classifier was trained with three cost parameter candidates [0.1, 1, 10], which control the trade-off between margin maximization and the tolerance of misclassification rate in the training step, and the best parameter was chosen by a grid search in nested cross-validations. The outlier boundary and cost parameter candidates were selected based on the estimated computational load and the documents of tools employed. Specifically, we here adopted a relatively small set of parameter candidates because of the constraint of the high computational load of searchlight analysis. Finally, the trained classifier predicted category or subcategory of seen source code from the leave-out test data and decoding accuracy was calculated as a ratio of correct-classifications out of all-classifications. Note that corrected misclassification cost weights were used in subcategory decoding to compensate for the imbalanced number of exemplars across subcategory classes.

The training and evaluation procedures were performed independently for each subject and a whole-brain decoding accuracy map was obtained per subject. We then conducted second-level analyses to examine the significance of decoding accuracies and the correlations between individual decoding accuracies and behavioral performances. For this purpose, the decoding accuracy maps were spatially smoothed using a Gaussian kernel of 6-mm full-width at half maximum (FWHM) and submitted to random effects analysis as implemented in SPM12. The analysis tested the significance of group-level decoding accuracy and Pearson’s correlation coefficient between individual decoding accuracies and behavioral performances. A relatively strict statistical threshold of voxel-level *p* < 0.05 familywise error (FWE)-corrected was used for decoding accuracy tests and a standard threshold of voxel-level *p* < 0.001 uncorrected and cluster-level *p* < 0.05 FWE-corrected was used for correlation tests. The chance-level accuracy (25% in category decoding and 9.72% in subcategory decoding; adjusted for imbalanced numbers of exemplar) and zero correlation were adopted as null hypotheses. Additionally, the resultant significant searchlight maps, i.e., decoding accuracy map and correlation map to behavioral performance, were superimposed on a single cortical surface of the ICBM152 template brain using BrainNet viewer (Xia et al., 2013). We performed this superimposition to identify the searchlight centers that had both sufficient information to represent functional categories of source code and significant correlation between individual behavioral performances and decoding accuracies.

### Data and code availability

The experimental data and code used in the present study are available from our repository: https://github.com/Yoshiharu-Ikutani/DecodingCodeFromTheBrain.

## Results

### Behavioral data

We evaluated the relationship between the adopted reference of programming expertise and behavioral performance on the program categorization task. A significant correlation was observed between AtCoder rate [mean = 954.3, SD = 864.6] and behavioral performance in the fMRI experiments [mean = 76.0, SD = 13.5 (%)], *r* = 0.593, *p* = 0.0059, *n* = 20 (Fig. 2*A*). The correlation remained significant if we included behavioral performances of non-rate-holders (i.e., novices) as zero-rated subjects; *r* = 0.722, *p* = 0.000007, *n* = 30. We additionally found a positive correlation between AtCoder rate and behavioral performance on subcategory categorization in the post-MRI experiments [mean = 65.9, SD = 17.0 (%)], *r* = 0.688, *p* = 0.0008, *n* = 20 (Fig. 2*B*). The significant correlation also remained significant if we included non-rate-holder subjects; *r* = 0.735, *p* = 0.000004, *n* = 30. From all behavioral data, we certainly concluded that behavioral performances on the program categorization task significantly correlated with expertise of competitive programming. The behavioral evidence allowed us to study the potential association between experts’ outstanding performances and brain activity patterns measured using fMRI while subjects performed this laboratory task.

### Multivoxel activity patterns associated with programming expertise

We first examined where we could decode the functional categories of source code from programmers’ brain activity. Figure 3 visualizes the searchlight centers that showed significantly higher decoding accuracy than chance as estimated from all subject data using a relatively strict whole-brain statistical threshold (voxel-level *p* < 0.05 FWE-corrected). The figure shows that significant decoding accuracies were observed in the broad areas of the bilateral occipital cortices, parietal cortices, posterior and ventral temporal cortices, as well as the bilateral frontal cortices around IFGs. Given the result, we confirmed that functional categories of source code were represented in the widely distributed brain areas and the cortical representations of each category class were linearly separable by a simple SVM classifier.

**Figure 4.**
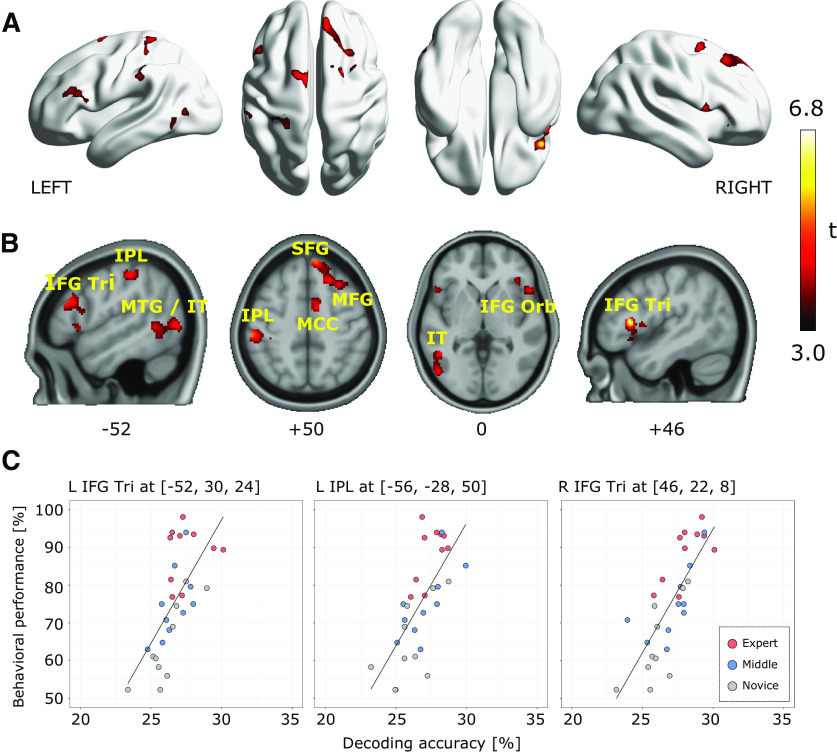
Searchlight-based correlation analysis between behavioral performances and decoding accuracies. ***A***, Locations of searchlight showing significant correlations. Significance was determined by a threshold of voxel-level *p* < 0.001 and cluster-level *p* < 0.05, FWE corrected for the whole brain. ***B***, Slice-wise visualizations of the significant clusters using bspmview (http://www.bobspunt.com/software/bspmview). ***C***, Correlation between behavioral performance and decoding accuracy. Each dot represents an individual subject data. For all significant clusters and peak correlations, see Table 2. SMG, supramarginal gyrus; IPL, inferior parietal lobule; MTG, middle temporal gyrus; IT, inferior temporal gyrus; SFG, superior frontal gyrus; MFG, middle frontal gyrus; IFG Tri, inferior frontal gyrus pars triangularis; IFG Orb, inferior frontal gyrus pars orbitalis; MCC, medial cingulate cortex.

To associate the cortical representation of source code with individual programming expertise, we investigated a linear correlation between behavioral performances and decoding accuracies for each searchlight location. Figure 4*A* visualizes the searchlight centers that showed significantly high correlation coefficients using thresholds of voxel-level *p* < 0.001 uncorrected and cluster-level *p* < 0.05 FWE-corrected. We observed significant correlations in the areas of bilateral IFGs pars triangularis (IFG Tri), right superior frontal gyrus (SFG), left IPL, left MTG and inferior temporal gyrus (IT); see the slice-width visualization shown as Figure 4*B* and Table 2 for the list of significant clusters. In this correlation analysis, the right IFG Tri showed the highest peak correlation coefficient. These results provided evidence that cortical representations in the distinct brain areas mainly located in frontal, parietal, and temporal cortices were significantly associated with experts’ outstanding behavioral performances on the program categorization task. In contrast, cortical representations in the bilateral occipital cortices including early visual areas did not show a significant correlation to individual behavioral performances, while significant decoding accuracies were broadly observed in the cortices shown as Figure 3.

**Table 2:** Clusters showing significant correlations between behavioral performance and category decoding accuracy (voxel-level *p* < 0.001 and cluster-level *p* < 0.05, FWE-corrected)

	MNI coordinates					
Region name	*x*	*y*	*z*	Peak corr. (*r*)	*t* value	Cluster extent
R IFG (Tri)	46	22	8	0.789	6.81	369
L posterior-medial frontal	−12	0	66	0.711	5.36	298
R superior medial gyrus	6	52	42	0.699	5.17	587
L IPL	−56	−28	50	0.698	5.16	649
R SFG	24	4	60	0.675	4.84	428
L IFG (Tri)	−52	30	24	0.671	4.79	346
L IT	−50	−54	0	0.635	4.35	347

Region names were identified using automated anatomic labeling atlas 2 (Rolls et al., 2015).

**Figure 5.**
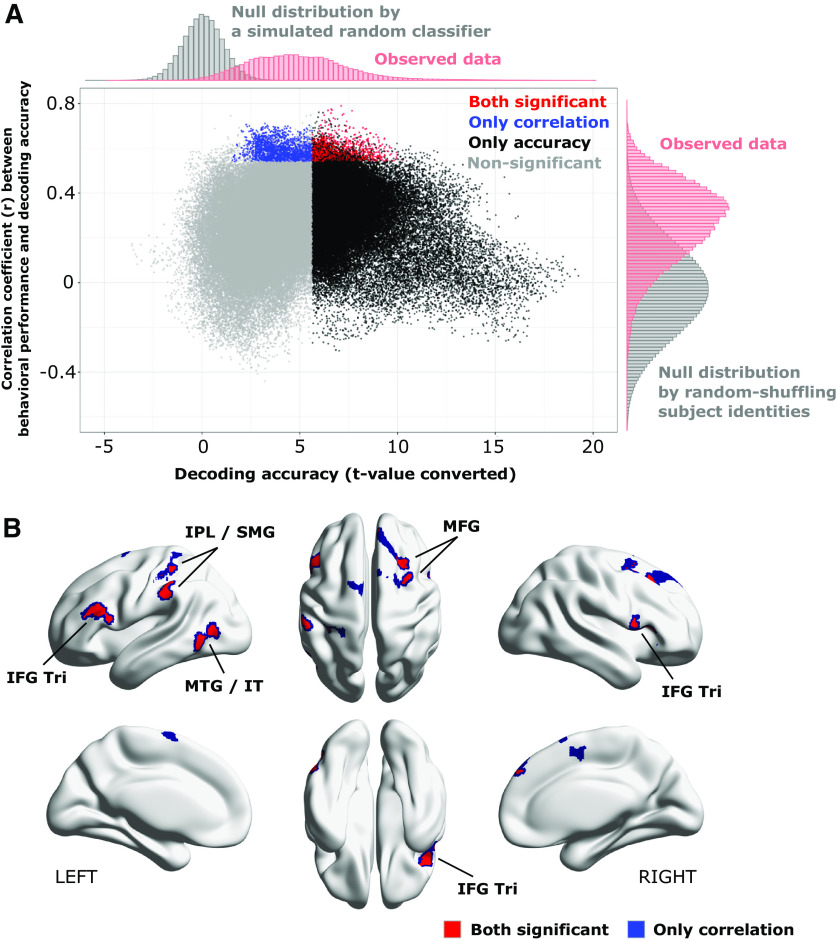
Identifying searchlight centers that showed both significant decoding accuracy and significant correlation to individual behavioral performances. ***A***, Scatter plot of searchlight results. *x*-axis shows *t* values calculated from all subjects’ decoding accuracies on each searchlight locations. *y*-axis indicates correlation coefficients between decoding accuracies and behavioral performances. Red-colored dots denote the searchlights showing both significant decoding accuracy and correlation, while blue and black denote those only showed significant decoding accuracy or correlations. Non-significant searchlights were colored in gray. The observed distributions of decoding accuracies and correlations are respectively shown on top-side and right-side of the figure accompanied with null distributions calculated by randomized simulations. ***B***, Locations of searchlight centers that showed both significant decoding accuracy and significant correlations to individual behavioral performances.

Two previous analyses separately showed where significant decoding accuracies exist and whether the decoding accuracies significantly correlate with behavioral performances. To achieve more validated evidence for the cortical representations associated with programming expertise, we integrated these two analyses and identified searchlight centers that had sufficient information to represent functional categories of source code and their decoding accuracies significantly correlated with individual behavioral performance. Specifically, the two significant searchlight maps, i.e., decoding accuracy map and correlation map to behavioral performance, were superimposed on a single cortical surface to investigate the overlap between them. As a result, we found 1205 searchlight centers (equal to 0.79%) that survived from both statistical thresholds of decoding accuracy and correlation to behavioral performances; shown as red-colored dots in Figure 5*A*. The survived searchlight centers were mainly observed in the bilateral IFG Tri, left IPL, left supramarginal gyrus (SMG), left MTG/IT, and right MFG as shown in Figure 5*B*. These results revealed a tight association between superior behavioral performances of expert programmers and improvement of decoding accuracy in these seven brain regions.

### Cortical representations of subcategory information

We next investigated where we could decode the subcategory of source code from programmers’ brain activity to examine finer-level cortical representations. In our experiment, subjects responded sort when they had been presented with the code snippets implementing one of three different sorting algorithms; i.e., bubble, insertion, and selection sorts (Fig. 1*A*). This cognitive process could be considered as a generalization process that incorporates different but similar algorithms (subcategory) into a more general functionality class (category). Additionally, several psychologists indicated that experts specifically show high behavioral performances in subordinate-level categorizations as well as basic-level categorizations (Tanaka and Taylor, 1991). In fact, we have observed that the ability to differentiate subcategory classes significantly correlated to programming expertise in competitive programming (Fig. 2*B*). This observation implies that the detailed difference of source code functionalities might be represented in programmers’ brain activity patterns. The decoding accuracy of subcategory may be correlated with programming expertise, although they classified only category classes, not subcategory, of given code snippets and the existence of subcategory classes had never been revealed until the end of the fMRI experiment.

**Figure 6.**
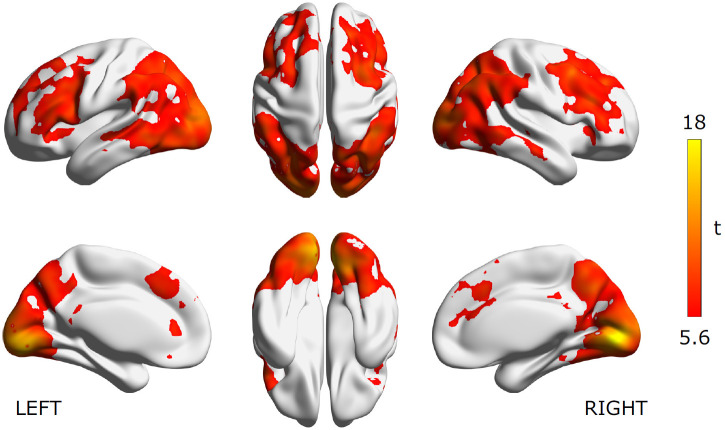
Decoding accuracy for subcategory of source code. Searchlight locations showing significant subcategory decoding accuracy than chance estimated from all subject data (*N* = 30). Heat colored voxels denote the centers of searchlights with significant subcategory decoding accuracy (voxel-level *p* < 0.05, FWE corrected). For the distribution of voxel-level peak subcategory decoding accuracies, see Extended Data Figure 6-1.

**Figure 7.**
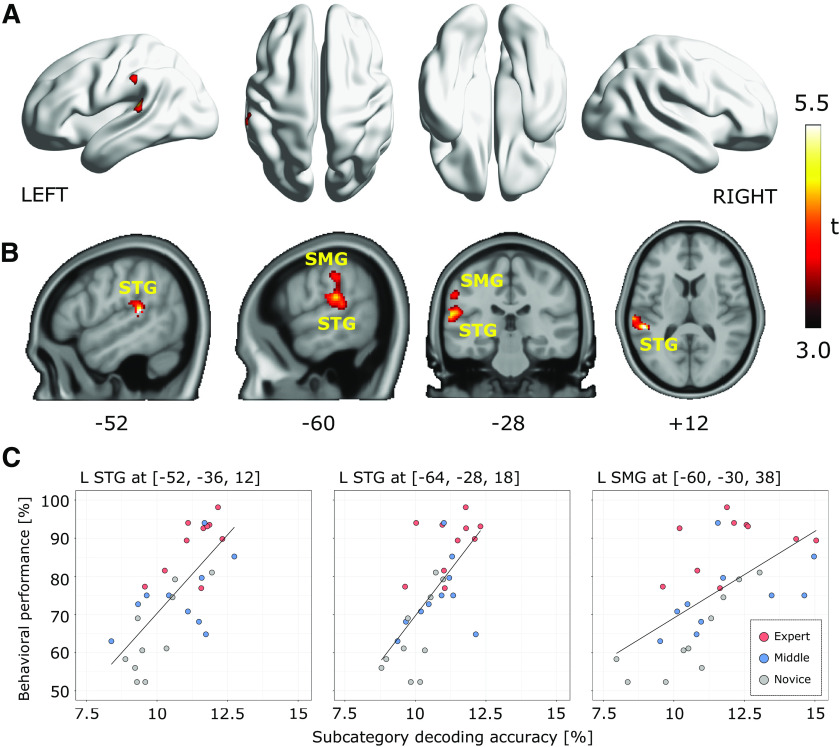
Searchlight-based correlation analysis between behavioral performances and subcategory decoding accuracies. ***A***, Locations of searchlight showing significant correlations. Significance was determined by a threshold of voxel-level *p* < 0.001 and cluster-level *p* < 0.05, FWE corrected for the whole brain. ***B***, Slice-wise visualizations of the significant clusters. ***C***, Correlation between behavioral performance and decoding accuracy. Each dot represents an individual subject data. Only one cluster (extent = 501 voxels) had significant correlation in this analysis and three peak correlations in the cluster were shown here. STG, superior temporal gyrus.

**Figure 8.**
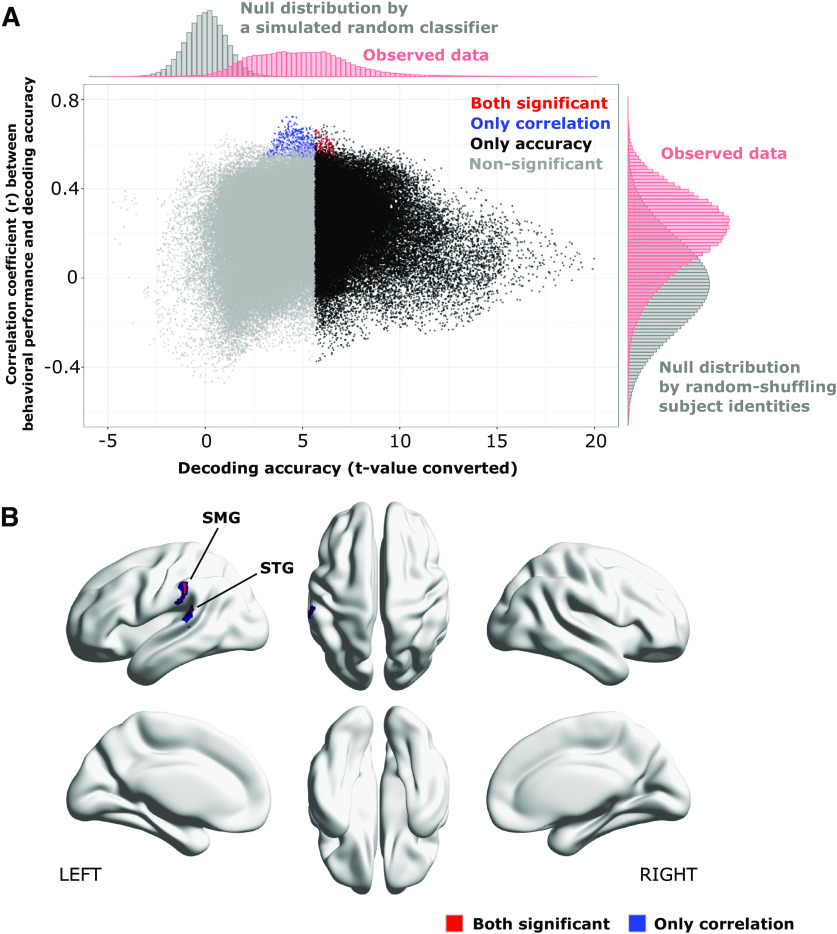
Identifying searchlight centers that showed both significant subcategory decoding accuracy and significant correlation to individual behavioral performances. ***A***, Scatter plot of searchlight results; *x*-axis shows *t* values calculated from all subjects’ decoding accuracies on each searchlight locations; *y*-axis indicates correlation coefficients between subcategory decoding accuracies and behavioral performances. Red-colored dots denote the searchlights showing both significant decoding accuracy and correlation, while blue and black denote those only showed significant decoding accuracy or correlations. Non-significant searchlights were colored in gray. The observed distributions of subcategory decoding accuracies and correlations are respectively shown on top-side and right-side of the figure accompanied with null distributions calculated by randomized simulations. ***B***, Locations of searchlight centers that showed both significant subcategory decoding accuracy and significant correlations to individual behavioral performances.

We employed searchlight analysis with the same setting as used in the previous analysis to reveal the spatial distribution of significant subcategory decoding accuracies and significant correlations to behavioral performances. Figure 6 illustrates the searchlight centers that showed significantly higher subcategory decoding accuracy than chance (9.72%; corrected for imbalanced exemplars) using a threshold of voxel-level *p* < 0.05 FWE-corrected. The linear correlation between subcategory decoding accuracies and individual behavioral performances was then assessed using thresholds of voxel-level *p* < 0.001 uncorrected and cluster-level *p* < 0.05 FWE-corrected (Fig. 7). As a result, only a cluster on the left SMG and STG showed a significant correlation; the peak correlation coefficient was observed in the left STG. Finally, we integrated the results from decoding and correlation analysis of subcategory and confirmed that 120 searchlight centers (equal to 0.08%) on the left SMG and STG survived from both statistical thresholds of decoding accuracy and correlation to behavioral performances (Fig. 8*A*, red-colored dots). These results suggest that cortical representations of fine functional categories on the left SMG and STG may play an important role in achieving advanced-level programming expertise, although the representations are not explicitly required by the tasks.

## Discussion

We have shown that functional categories of source code can be decoded from programmers’ brain activity measured using fMRI. Decoding accuracies on the bilateral IFG Tri, left IPL, left SMG, left MTG and IT, and right MFG were significantly correlated with individual behavioral performances on the program categorization task. Furthermore, decoding accuracies of subcategory on the left SMG and STG were also strongly correlated with the behavioral performances while the subordinate-level representations were not directly induced by the performing tasks. Our results revealed an association between the outstanding performances of expert programmers and domain-specific cortical representations in these brain areas widely distributed in the frontal, parietal, and temporal cortices.

Previous fMRI studies on programmers have aimed at characterizing how programming-related activities, such as program comprehension and bug detection, take place in the brain (Siegmund et al., 2014, 2017; Floyd et al., 2017; Castelhano et al., 2019; Peitek et al., 2018a,b). Exceptionally, an exploratory study reported that BOLD signal discriminability between code and text comprehension was negatively correlated with participants’ GPA scores in a university (Floyd et al., 2017). However, the relationship between GPA scores and programming expertise was ambiguous and the observed correlation was relatively small (*r* = −0.44, *p* = 0.016, *n* = 29). Our aim in the present study was substantially different: we sought the neural bases of programming expertise that contribute to expert programmers’ outstanding performances. To address the goal, we adopted an objectively-determined reference of programming expertise and recruited a population of subjects covering a wide range of programming expertise. Despite the difference in research aims, a subset of brain regions specified in this study was similar to those specified by prior fMRI studies on programmers (Siegmund et al., 2014, 2017; Peitek et al., 2018a). In particular, this study associated the left IFG, MTG, IPL, and SMG with programming expertise, while previous studies related them with program comprehension processes. This commonality may suggest that both program comprehension processes and its related expertise depend on the same set of brain regions.

The potential roles of the specified brain regions in our study should be addressed to orient future researches on programming activity and expertise. First, the left IFG Tri and the left posterior MTG are frequently involved in semantic selecting/retrieving tasks (Démonet et al., 1992; Thompson-Schill et al., 1997; Simmons et al., 2005; Price, 2012). Several studies indicated that these two regions are sensitive to cognitive demands for directing semantic knowledge retrieval in a goal-oriented way (Rodd et al., 2005; Kuhl et al., 2007; Whitney et al., 2011). The involvement of the two regions in our findings may be induced by similar demands specialized for the retrieval of program functional categories and suggest that higher programming expertise is related to the abilities of goal-oriented knowledge retrieval. Second, many neuroscientists have shown the left IPL and SMG to be functionally related to visual word reading (Bookheimer et al., 1995; Philipose et al., 2007; Stoeckel et al., 2009) and episodic memory retrieval (Wagner et al., 2005; Vilberg and Rugg, 2008; O'Connor et al., 2010). Both cognitive functions potentially relate to the program categorization task used in our experiment. Visual word reading can be naturally engaged since source code is comprised of many English-like words and subjects may have actively recollected previously-acquired memories to compensate for insufficient clues because they had only 10 s to categorize the given code snippet. The involvements of the left IPL and SMG in programming expertise suggest that expert programmers might possess different reading strategies and/or depend more on domain-specific memory retrieval than novices. In addition, the set of IFG and IPL has been frequently discussed together as a fronto-parietal network and they often show synchronous activity in a wide range of tasks (Watson and Chatterjee, 2012; Ptak et al., 2017). Importantly, a recent fMRI study on programmers suggested an association between program comprehension and fronto-parietal network that was functionally related to formal logical inference (Liu et al., 2020). Our results are consistent with these findings, implying that the fronto-parietal network plays a key role in experts’ program comprehension processes.

Other novel findings in the present study included potential involvement of the left IT, right MFG, and right IFG Tri with programming expertise. Importantly, these regions were not specified by previous studies focusing on the relationship between brain activity and program comprehension processes of non-expert subjects (Siegmund et al., 2014, 2017; Floyd et al., 2017; Peitek et al., 2018a), suggesting that the regions might be more related to expert programmers’ program comprehension processes. Because the left IT is well known for the function in high-level visual processing including word recognition and categorical object representations (Chelazzi et al., 1993; Nobre et al., 1994; Kriegeskorte et al., 2008); our results may suggest that the high-level visual cortex in expert programmers could be fine-tuned by their training experience to realize faster program comprehension process. From another perspective, the observed map involving the left IFG Tri, IPL, and MTG/IT (Fig. 4*A*) could be associated with a semantic system in the brain (Patterson et al., 2007; Binder et al., 2009). Our results might suggest that an expert programmer’s brain recruits a similar language-related network for both natural language processing and program comprehension. In contrast, the primary visual area showed significant decoding accuracy but no correlation to programming expertise. The primary visual area mainly reflects primitive visual features such as color, contrast, spatial frequency (Tong, 2003), while computations in the high-level visual cortex are characterized by both bottom-up (i.e., how stimuli are visually represented) and top-down (how the representation is used for a cognitive task) effects (Kay and Yeatman, 2017). Previous studies indicated that fine-tuned representations in the high-level visual cortex, rather than in the primary visual area, could be associated with visual expertise (Bilalić et al., 2016) and reading skill Kubota et al. (2019). In our experiment, the primary visual area represented a large amount of visual information regardless of programming expertise levels because all subjects were presented with the same set of code snippets inducing similar visual patterns on their retinas. Therefore, the information in the primary visual area was sufficient to decode category and subcategory classes but the decoding accuracies were not necessarily to be correlated with individual behavioral performances. Meanwhile, the amount of information represented in the high-level visual cortex might be modulated by individual programming expertise. In line with previous expertise studies, our results imply that expertise in program comprehension could be mainly associated with high-level visual perception.

The right MFG and IFG Tri are functionally related to stimulus-driven attention control (Corbetta et al., 2008; Japee et al., 2015). The involvement of these two regions suggests that programmers with high-level programming expertise may employ different attention strategies than less-skilled ones. Moreover, additional engagements of right hemisphere regions in experts are common across expertise studies. For example, chess experts (Bilalić et al., 2011) and abacus experts (Tanaka et al., 2002; Hanakawa et al., 2003) showed additional right hemisphere region involvements when performing their domain-specific tasks. Several fMRI studies further suggest that such activation shifts from left to right hemisphere may be related to experts’ cognitive strategy changes (Bilalić et al., 2011; Tanaka et al., 2012). Cognitive strategy changes have been observed repeatedly in comparisons between expert and novice programmers: a major characteristic is a transition from bottom-up (or textual-driven) to top-down (or goal-driven) program comprehension, which becomes feasible by experts’ domain-specific knowledge (Koenemann and Robertson, 1991; Fix et al., 1993; Von Mayrhauser and Vans, 1995). The involvement of the right MFG and IFG Tri observed in this study might be related to such cognitive strategy differences between programmers in the program categorization task. From another perspective, activations in the prefrontal and parietal regions including bilateral IFG/MFG and left IPL have been associated with the extent of cognitive demands (Harvey et al., 2005). While our study did not have a direct indicator of cognitive demands across categories, the difference in behavioral performances for each category can be a clue to assess the extent of cognitive demand across the categories. We used the one-way ANOVA to test the difference in mean behavioral performances between categories but no significant difference was found for any groupings (see Extended Data Fig. 2-1). Although these results do not provide a direct indication of cognitive demands across categories, we have no positive evidence that the extent of cognitive demands had a significant effect on the observed decoding accuracies.

10.1523/ENEURO.0405-20.2020.f2-1 Extended Data Figure 2-1 Behavioral performance of each category in the fMRI experiment. Numerics from 3rd (Math) to last columns denote mean ± SD. One-way ANOVA found no significant difference in behavioral performances between categories for any groupings (expert, *F*_(3,36)_ = 1.38, *p* = 0.27; middle, *F*_(3,36)_ = 2.99, *p* = 0.06; novice, *F*_(3,36)_ = 2.81, *p* = 0.07; all, *F*_(3,116)_ = 2.02, *p* = 0.12). Download Figure 2-1, XLSX file.

10.1523/ENEURO.0405-20.2020.f3-1 Extended Data Figure 3-1 Box plots of the voxel-level peak category decoding accuracies on several brain regions. Each dot represents decoding accuracy of individual subject. The dashed line indicates chance-level accuracy (25%). SMG, supramarginal gyrus; IPL, inferior parietal lobule; MTG, middle temporal gyrus; IT, inferior temporal gyrus; IFG Tri, inferior frontal gyrus pars triangularis. Download Figure 3-1, EPS file.

10.1523/ENEURO.0405-20.2020.f6-1 Extended Data Figure 6-1 Box plots of the voxel-level peak subcategory decoding accuracies on several brain regions. Each dot represents decoding accuracy of individual subject. The dashed line indicates chance-level accuracy (9.72%). SMG, supramarginal gyrus; IPL, inferior parietal lobule; MTG, middle temporal gyrus; IT, inferior temporal gyrus; IFG Tri, inferior frontal gyrus pars triangularis. Custom software code. The custom MATLAB code used for the decoding analysis in the paper. Download Figure 6-1, EPS file.

Our results associated programming expertise with decoding accuracies of not only category but also subcategory, although the subordinate-level categorizations were not explicitly required by the performing task. We observed that individual behavioral performances were significantly correlated with subcategory decoding accuracies on the left STG and SMG. These two regions are functionally related to prelexical and phonological processing in natural language comprehension (Démonet et al., 1992; Moore and Price, 1999; Burton et al., 2001). Interestingly, we also found a significant correlation between behavioral performances and category decoding accuracies on the temporal regions (left MTG and IT) associated with more semantical processing (Rodd et al., 2005; Whitney et al., 2011; Price, 2012). If these functional interpretations could be adaptable to program comprehension processes, it would be intuitive that subordinate concrete concepts (i.e., subcategory) of source code are processed in the left STG/SMG and more semantically abstract concepts (i.e., category) are represented in the left MTG/IT. Further, Mkrtychian et al. (2019) have associated STG, MTG, and IFG with the processing of abstract concepts in their review on concreteness effects, implying that representations in these three regions could reflect relative differences in abstractness between the category and subcategory in our study. These interpretations might suggest a hypothesis that an expert programmer’s brain has a hierarchical semantic processing system to obtain mental representations of source code for multiple levels of abstraction.

Our decoding framework specialized for the functional category of source code could be extended by the recent advances of decoding/encoding approaches in combination with distributed feature vectors (Diedrichsen and Kriegeskorte, 2017). Several researchers have demonstrated frameworks to decode arbitrary objects using a set of computational visual features representing categories of target objects (Horikawa and Kamitani, 2017) and to decode perceptual experiences evoked by natural movies using word-based distributed representations (Nishida and Nishimoto, 2018). Other studies have also used word-based distributed representations to systematically map semantic selectivity across the cortex (Huth et al., 2016; Pereira et al., 2018). Meanwhile, researchers in the program analysis domain have proposed distributed representations of source code based on abstract syntax tree (AST; Alon et al., 2019a; Zhang et al., 2019). Alon et al. (2019b), for instance, have presented continuous distributed vectors representing the functionality of source code using AST and path-attention neural network. The combination of recent decoding/encoding approaches and distributed representations of source code may enable us to build a computational model of program comprehension that connects semantic features of source code to programmers’ perceptual experiences.

### Limitations of the study

The results obtained via the present study were limited to a specific type of programming expertise evaluated by the expertise reference and laboratory task used in the experiment. We particularly examined the ability to semantically categorize source code that correlated with programming expertise to win high scores in competitive programming contests. Perhaps there is a qualitative gap between expertise in competitive programming and practical/industrial software development. For example, the ability to write efficient SQL programs may be an explicit indicator of another type of programming expertise, but this study did not cover such type of programming expertise. The program categorization task used in this study primarily evaluated the skill in recognizing algorithms quickly and accurately, which was one aspect of a wide range of cognitive skills that constitute programming expertise. We considered that the evaluated skill is related to program comprehension and is also connected to skills in code refactoring and debugging because these processes require a deep understanding of algorithms or how the code works, while its relation to writing code is not assessed in this study. Thus, our results should not be taken to imply the relationship between the neural correlates revealed here and other types of programming expertise that could not be examined by this experiment. However, it is also a fact that we cannot investigate the neural bases of programming expertise without a clear definition of expertise indicator and laboratory task that well fit the general constraints of fMRI experiments. To mitigate the potentially inevitable effects caused by this limitation, we adopted the objectively-determined reference of programming expertise that directly reflects programmers’ actual performances and recruited a population of subjects covering a wide range of programming expertise. This study can be a baseline for future researches to investigate the neural bases of programming expertise and related abilities.

Our experiment, which was designed to fit the general constraints of fMRI measurement, might embrace several caveats to external validity. First, we used the relatively small code snippets with 30 lines at maximum because of the constraint of the MRI screen size. Behavioral performances on system-level source code were not assessed in the study. Thus, generalizing our results to the expertise in systems-level program comprehension was not guaranteed. Second, only Java code snippets were used as experimental stimuli in this study. The results obtained via the experiments might be biased by the programming language selected, for example, Python has more natural-language-like syntax than Java and might induce more activation in language-related brain regions. While a recent fMRI study has examined brain activities elicited by code written in two programming languages (Python and ScratchJr; Ivanova et al., 2020), it is still unclear whether the choice of a specific programming language can alter an expert’s brain activity pattern. The relationship between programming expertise and types of programming languages (e.g., procedural vs functional languages) is expected to be examined in future work.

Another potential concern of the present study was the unfair gender balance in the subject population. While 95 programmers completed our entry questionnaire to be registered as candidate subjects, only one middle-level woman candidate and zero woman expert were found (see Materials and Methods, Subjects). From this situation, we recognized the unavoidable gender bias in our target population. To properly cover a wide range of programming expertise, we were forced to give up on maintaining gender balance at each expertise level. However, several fMRI studies have reported possible gender differences in behavior, cognitive function, and neuroimaging data (David et al., 2018; Huang et al., 2020). The results obtained via this study might be biased by the gender imbalance of the subject population. Future work should investigate whether behavioral and cognitive differences would be found between man and woman programmers. In addition, while our sample size was determined in line with previous expertise studies, ten subjects for each expertise level was not a big population and were insufficient to show statistically significant results between different expertise classes. Therefore, making mention of comparison between novice-middle or middle-expert must be with great caution. Larger samples would be desirable in future replication or follow-up studies.

Our findings reveal an association between programming expertise and cortical representations of program source code in a programmer’s brain. We demonstrated that functional categories of source code can be decoded from programmer’s brain activity and the decoding accuracies on the seven regions in the frontal, parietal, and temporal cortices were significantly correlated with individual behavioral performances. The results additionally suggest that cortical representations of fine functional categories (subcategory) on the left SMG and STG might be associated with advanced-level programming expertise. Although research on the neural basis of programming expertise is still in its infancy, we believe that our study extends the existing human expertise literature into the domain of programming by demonstrating that top-level programmers have domain-specific cortical representations.

## Synthesis

Reviewing Editor: Macià Buades-Rotger, University of Luebeck Decisions are customarily a result of the Reviewing Editor and the peer reviewers coming together and discussing their recommendations until a consensus is reached. When revisions are invited, a fact-based synthesis statement explaining their decision and outlining what is needed to prepare a revision will be listed below. The following reviewer(s) agreed to reveal their identity: Norman Peitek.

Dear authors,

Thanks for your work on multivariate activity patterns underlying programming expertise. The paper reports a well-designed and conducted study with a novel experiment design. The authors tackle a highly relevant topic in software engineering, and the results are interesting for both the neuroscience and programming audiences. Overall, the submitted manuscript consists of sound, well-presented analyses. Both the reviewer and me -as co-reviewer and review editor- would support its publication after some refinements and clarifications. Note that the reviewer is aware of the authors’ identity due to familiarity with their work, albeit there is no conflict of interest. Also note that some of the links in the extended data break double-blind, which is not relevant in this case but is something to pay attention to in future submissions to journals with a double-blinded review format.

# Summary

The submitted paper reports on an fMRI study on programmers with different skill levels categorizing source code snippets. The authors invited participants (n=30 overall) and grouped them into different skill levels depending on an objective score on a coding challenge Web site. Participants categorized 72 source code snippets into categories in the fMRI scanner, and in subcategories later outside the fMRI scanner. A searchlight analysis decoding the observed brain activation found several activated brain areas in which an SVM classifier can distinguish snippet category and programmer expertise. The results show that expert programmers have a detectable difference in cortical representation of different categories of source code.

# Strengths & Weaknesses

Strengths:

+ solid empirical research with rigorous design

+ generally well-written and structured

+ important and highly relevant research topic

Weaknesses (see details below):

- lack of a deeper discussion on neural mechanisms to buttress the hypotheses

- missing perspective of the disconnect between the details of the presented study and its claims to programmer expertise

- room for improvement in the reporting

# Major points

- Lines 37-38: The authors speak of “left-lateralized brain regions", which is too vague for a neuroscience journal. Although the address this in depth in the Discussion, the Introduction would greatly benefit from a brief (though clear and specific) discussion of the neural mechanisms underlying programming expertise. Some questions to tackle are e.g. which regions are relevant for programming? What computations might these regions carry out? Which regions/computations are shared with other cognitive operations, which might be specific for programming?

- Lines 62-64: On a similar note as the previous point, the authors’ hypothesis is formulated too broadly: “Our core hypothesis is that higher programming expertise and experts’ outstanding performances relate to specific multi-voxel pattern representations, potentially influenced by their domain-specific knowledge and training experiences.”. This is equivalent to saying that the authors expect to find *some effect* in the brain, which, again, is highly unspecific. Where in the brain should multi-voxel representations differentiate between expert and non-expert programmers?

- The authors did a great job by using a fairly objective coding challenge score to separate the groups and identify programming experts. This seems much better than most state-of-the-art research simply using years of experience. However, I am curious why this was only done for the expert and mid-range group, but not all the way down for the novice group as well? In my mind, there are now three groups of: expert, mid-range, unknown. It seems more consistent to me to select novice participants based on *low* AtCoder scores instead of *no score*. Figure 2 seems to indicate that there is quite a range of behavioral performance along the novice group. The decision to go with no scores should be explained in the paper.

- One further major weakness that should be addressed in a revision: the presented study observes programmers in categorizing source code snippets, which is a logical choice and reasonable based on research in other domains. However, recognizing algorithms only appears to be one aspect of a wide range of skills that resembles programming expertise. Can we safely conclude that being an expert at recognizing algorithms also comes with great skill for comprehending source code, finding bugs, writing code, etc? The study and its data work on one single cognitive skill, but the paper argues mostly on a more abstract level of general expertise (e.g., the title of the paper simply says “expert programmers” with no hint toward the limitation of the observed task). There needs to go more work into linking the given task with abstract programming expertise, or more accurate discussion of the observed expertise. Please clarify.

- Many studies in software engineering investigate expertise with a binary approach, that is, novice versus expert. I really like that the authors designed a study that did not only look at extremes, but also included an in-between group. However, in the introduction the motivation for the presented study is entirely on novice/expert. I think a quite critical topic is to understand how “mid-range” programmers think, not just experts (that by nature are, at most, 10% of the population). I think the introduction could be stronger if you motivate your work that your study not only looks at novice and experts, but also at a “mid-range” programmers. Similarly, the discussion could pick up on this range of skill levels again. Do mid-range programmers in contrast to your novices already have a fine-tuned cortical representation, too? This is interesting and should not be skipped!

- Similarly, does expertise on small code snippets extrapolate to skill on a systems level? Can a company select the best programmers from an applicant pool by giving them a snippet categorization task? There needs to be a discussion on the external validity of the presented study.

- In L43-45, the authors discuss that a study by Floyd et al. used GPA as an expertise indicator, but GPA may include other factors such as IQ, memory, calculation. Is it possible that expert programmers are experts due to these skills? Or that developing programming expertise trains these mental abilities? As far as I am aware, there is no research on this topic, and it seems a bit bold to claim that they are disconnected. Please clarify or provide references.

- This paper will attract quite a bit of interest from a software engineering audience. Thus, it would be worth-while to make this paper as accessible as possible for readers without a neuroscience background (even if it is in a neuroscience journal). For example, in the introduction in L62-69 the authors discuss how there has been neuroscience research investigating expertise in other domains. I think it would be very valuable to pick one or two studies and demonstrate in more detail how expertise shows itself in those domains and how understanding expertise helps this domain forward.

- Lines 109-110: the acronyms LOC and CPL need not be used, as these words are used only once more in the text. Please just write what they stand for.

- In L117 and L126, the authors describe that they removed semantic information and syntax highlighting from the source code, effectively inducing bottom-up comprehension. This is a rather curious decision, since research indicates that increased expertise enables programmers to apply top-down comprehension, while novices tend to be stuck in bottom-up comprehension. Would removing semantic information and syntax highlighting not remove some of the delta between experts and novices?

- In L179-194, the authors describe their analysis in more detail. How did you select the parameters, for example, the outlier reduction or cost parameters? Also, please briefly describe what the cost parameters are, and spell out the acronym SVM because this is non-standard for users unfamiliar to classification algorithms.

- Line 205: how was the adjustment for imbalanced number of exemplars exactly performed?

- Lines 239-241 and 278-270, Figures 4 and 7: please delete the r- and p-values associated with the brain-behavior correlations. These effects have been shown to be, by necessity, inflated because they would not otherwise survive whole-brain correction, and reporting them constitutes a circular analysis or “double-dipping", see https://www.nature.com/articles/nn.2303

- Lines 247-257: how exactly were the two analyses (decoding and decoding-performance correlation) merged? Was it a conjunction analysis? Please detail the exact procedure in the Methods section and mention it here.

- Lines 288-289: please spell out the acronyms for the brain regions, as some readers might skip straight to the first paragraph of the Discussion for a results summary.

- The finding that programming categories could be decoded from low-level visual regions but that decoding accuracy did *not* correlate with programming expertise should be discussed in greater detail.

- On a related note, the authors discuss the role of each brain in isolation, which is acceptable because the study only addresses local patterns of activity. However, some of the identified brain regions such as the IPL and IFG belong to the same fronto-parietal network and they often show synchronous activity in a wide range of tasks.

- In L331 the authors discuss that program comprehension and programming expertise are different, but this does not seem obvious to me. Does expertise not play a role in how programmers comprehend source code?

- Lines 409-417: the authors use sex and gender indistinguishably in this paragraph. As the sample was comprised of human participants, it would be preferrable if the authors stick to the term “gender” and the terms “woman/women” and “man/men” rather than “male” or “female” throughout the manuscript, also in the Subjects section (which should be labelled “Participants” rather than “Subjects” for the same reason).

- Indeed, even if the sample size was in line with previous studies, larger samples would be desirable in future replication or follow-up studies. This should be mentioned in the Limitations.

- Lines 419-420: Please remove the word “tight", as this is a pilot study with a small sample and thus the authors should interpret effect sizes with due caution.

- I am unfamiliar with AtCoder, but with the coding Web sites I know, the score often represents more effort rather than pure skill. For example, someone trying to solve challenges for 20 hours may get a higher score than a higher skilled person trying for 3 hours. Is there potential for such problem with AtCoder as well? If so, this should be discussed in the study limitations.

- Figures 4 and 7 show decoding accuracy of around 25-28% and 8-12%, respectively. Is that not quite low and almost at a baseline of guessing? Please clarify.

- Please share more details on the post-session of subcategory identification. For example, in L149, the authors write “unanswered trials”. Was there a time limit? Was this done at a computer or on paper?

- There are more recent fMRI studies in software engineering that may be worth discussing:

Biases and Differences in Code Review using Medical Imaging and Eye-Tracking: Genders, Humans, and Machines, Huang, Yu and Leach, Kevin and Sharafi, Zohreh and McKay, Nicholas and Santander, Tyler and Weimer, Westley (FSE 2020)

Comprehension of computer code relies primarily on domain-general executive resources, A. Ivanova, S. Srikant, Y. Sueoka, Hope H. Kean, R. Dhamala, Una-May O’Reilly, M. U. Bers, E. Fedorenko (2020)

Computer code comprehension shares neural resources with formal logical inference in the fronto- parietal network, Liu, Y., Kim, J., Wilson, C., Bedny, M. (2020)

## Minor Points

L3: “Many have shown” -> “Many studies ...”?

L35: “functional MRI” -> functional magnetic resonance imaging

L132: “randomized” -> the described order is “pseudo-randomized"

L137-138: “The training session was performed within ten days before the fMRI experiment to mitigate potential noises caused by task unfamiliarity”. “Confounds” would more appropriate than “noises” in this context.

L215/219: “correlation was kept” -> “correlation remains significant”?

L261: the form “they” in plural form is preferrable to “he/she"

L383: “visual futures” -> features?

Figure 1: Could you please explain the unit of each column? Is programming experience (PE) in years?

Figure 2: Could you separate the “With AtCode-rate” into your two categories of “experts” and “mid-range” with different colors?
